# Rapid Detection of *Philaenus italosignus* Drosopoulos & Remane, 2000 (Hemiptera: Aphrophoridae) with Real-Time PCR Probe LNA Technology

**DOI:** 10.3390/insects16101014

**Published:** 2025-09-30

**Authors:** Domenico Rizzo, Alice Downes, Sara Campigli, Bruno Palmigiano, Claudia Gabriela Zubieta, Viola Papini, Michela Moriconi, Francesca Garganese, Ugo Picciotti, Aziza Husein, Chiara Ranaldi, Edson Bolige, Linda Bartolini, Francesco Porcelli

**Affiliations:** 1 Laboratory of Phytopathological Diagnostics and Molecular Biology, Tuscany Regional Plant Health Service, Via Ciliegiole 99, 51100 Pistoia, Italy; domenico.rizzo@regione.toscana.it (D.R.); a.downes@studenti.unipi.it (A.D.); bruno.palmigiano@regione.toscana.it (B.P.); claudiagabriela.zubieta@regione.toscana.it (C.G.Z.); violapapini@gmail.com (V.P.); michmoriconi@gmail.com (M.M.); chiara.ranaldi16@gmail.com (C.R.); edson.bolige@gmail.com (E.B.); linda.bartolini@regione.toscana.it (L.B.); 2 Department of Agriculture, Food, and Environment (DAFE), University of Pisa, Via del Borghetto 80, 56124 Pisa, Italy; 3 Department of Agriculture, Food, Environment and Forestry (DAGRI), University of Florence, Piazzale delle Cascine 28, 50144 Florence, Italy; sara.campigli@unifi.it; 4 Department of Soil, Plant and Food Sciences (DiSSPA), University of Bari Aldo Moro, Via G. Amendola 165/A, 70126 Bari, Italy; francesca.garganese@uniba.it (F.G.); aziza.hussein@uniba.it (A.H.); francesco.porcelli@uniba.it (F.P.)

**Keywords:** biological invasion, alien, quarantine, invasive, plant pathogen, vector, spittlebug, Olive Quick Decline Syndrome, OQDS

## Abstract

To date, *Philaenus spumarius* (Linnaeus, 1758), *Philaenus italosignus* Drosopoulos & Remane, 2000, and *Neophilaenus campestris* (Fallén, 1805) are proven vectors of the phytopathogenic bacterium *Xylella fastidiosa* Wells et al., 1987 in Europe. Currently, the identification of these three species relies on the well-documented status of morphological and taxonomical characters, making the discrimination of vector adult males possible by genitalia comparison. This study updates the biomolecular diagnostic tests with a rapid identification tool for *P. italosignus*, using locked nucleic acid (LNA) probe technology. The test also overcomes the difficulties associated with the morphological identification of females and juveniles. The morphological α-taxonomic identification of the male, achieved through comparison with the type of the species, retains its primary role in specimen identification for probe building. Later, the proposed assay can contribute to the rapid identification of *P. italosignus* by the secondary (molecular) identification step. The new LNA qPCR test offers high reliability and reproducibility in the identification of *P. italosignus* instars, thus improving targeted surveys of *X. fastidiosa* vector populations and allowing discrimination between species collected in the field. The accurate identification and census of vector individuals, regardless of their gender and instar, enhances the efficacy of *Xylella* IPM-DSS (Integrated Pest Management Decision Support System) strategies.

## Simple Summary

This biomolecular diagnostic test for the rapid identification of *Philaenus italosignus* Drosopoulos & Remane, 2000, is valuable in identifying one of the hemipteran species involved in the Italian invasion of *Xylella fastidiosa* Wells et al., 1987. The test utilizes a locked nucleic acid (LNA) probe, which increases the probe’s affinity with the target, thus ensuring higher specificity. The new qPCR test with LNA probe has proven to be a more reliable and reproducible method for identifying the different instars of *P. italosignus*, thereby improving territorial surveys for *X. fastidiosa* vector population management strategies and allowing discrimination between species collected in the field.

## 1. Introduction

The vectors responsible for the spread of *Xylella fastidiosa* Wells et al., 1987 (Xf) [1] in Italy are indigenous xylem-sap-feeding Hemipterans. Despite all Aphrophoridae being potential vectors of *X. fastidiosa* [2], only *Philaenus spumarius* (L., 1758—the Meadow Spittlebug) has to date been found responsible for vectoring Xf in the field [3]. *Neophilaenus campestris* (Fallén, 1805) and *Philaenus italosignus* (Drosopoulos & Remane, 2000) are also considered vectors [4], although their role as vectors appears to be minimal [5,6] in the Italian pathosystems [7,8,9].

In the case of *P. italosignus*, molecular analyses using mitochondrial and nuclear DNA sequences confirm that the insect belongs to a clade comprising other species restricted to the Mediterranean. *Philaenus* spp. taxonomy and species descriptions use the male genitalia and anal tube segments [8]. Other Mediterranean *Philaenus* species are:

- *Philaenus arslani* Abdul-Nour & Lahoud, 1996, present in Lebanon [10], associated with *Echinops* spp., *Carduus* spp., *Cirsium* spp. (Asteraceae), and *Cistus* spp. (Cistaceae) [11];
- *Philaenus loukasi* Drosopoulos & Asche, 1991, is present in Greece [12] and is associated with *Eryngium* spp. (Asteraceae) [11];
- *Philaenus maghresignus* Drosopoulos and Remane, 2000, present in Morocco, Algeria, Spain, and Tunisia [13,14], feeds on *Asphodelus* spp. (Asphodelaceae) [11];
- *Philaenus philopotamos* Bückle & Guglielmino, 2025, recently described in north-east Italy [15], associated with *Myricaria germanica* (L.) Desv. (Tamaricaceae) and *Calamagrostis pseudophragmites* (Haller) Koeler (Poaceae) [15];
- *Philaenus signatus* Melichar, 1896, present in Greece [14], feeds on *Asphodelus* spp. (Asphodelaceae) [11];
- *Philaenus tarifa* Remane and Drosopoulos, 2001, which is present in the Iberian Peninsula [16], grows on *Asphodelus* spp. (Asphodelaceae) [11];
- *Philaenus tesselatus* Melichar, 1889, is present in Tunisia [17] associated with various dicotyledonous plants [11].

Morphological and phylogenetic data divide the Mediterranean *Philaenus* species into two groups: *P. italosignus*, *P. maghresignus*, *P. signatus*, and *P. tarifa* into the *signatus* group; while *P. arslani*, *P. loukasi*, *P. spumarius*, and *P. tesselatus* are in the *spumarius* group [18].

*Philaenus spumarius* differs from *P. italosignus* due to its morphological traits and ecological preferences [19,20]. *Philaenus spumarius* is a widespread Palearctic species that also thrives in regions as far afield as New Zealand, Hawaii, the United States, Canada, and Japan [21,22,23]. *Philaenus spumarius* prefers dicotyledonous plants and winter-cold continental climates, as suggested by its reproductive parapause [8].

*Philaenus italosignus* is endemic to central and southern Italy, where it is zonal in Udvardy’s Palearctic Mediterranean province [24]. However, *P. italosignus* does not exhibit significant vector competence, mostly thriving on *Asphodelus ramosus* L. 1753 (Asphodelaceae) [14,25], and feeding on olive, primarily under restricted conditions.

The interest in the adults, male and female, and their morph identification arises from the need to avoid overestimating the field population of *P. spumarius* due to morphological similarities between the two sympatric species. *Philaenus italosignus* inhabits several areas and niches in Italy, closely associated with the presence of *A. ramosus* [14], its host plant species [25]. The choice of *P. italosignus* for its Mediterranean monocot, fire-resistant, and perennial host plant [26,27] represents an interesting shift to monophagia in comparison to *P. spumarius’* vast polyphagia [28]. The choice allows *P. italosignus* to thrive in minute, diverse niches, permitting its presence in various Italian areas that are overgrazed or otherwise degraded [25,26,29]. However, *P. italosignus* is primarily associated with Italian regions [14]. In terms of phylogeography, the evidence suggests that *P. italosignus* differentiated in the Italian Mediterranean area, adapting to the climate and the availability of host plants during glacial periods. The genetic diversity observed within *P. italosignus* populations suggests that this species has adapted to niches different from those of *P. spumarius*, which may have facilitated its spread [30]. It is worth mentioning that, from the point of view of chromosome morphology recognition, *P. italosignus* exhibits significant heterochromatin C, with prominent bands on both autosomal and sex chromosomes. The variability in heterochromatin distribution suggests a significant evolutionary influence on the genetic diversity and adaptability of the species [31].

Adults of *Philaenus* spp. show dorsally a well-studied polymorphism on the head, prothorax, and tegmina [32,33,34,35,36,37,38,39]. The polymorphism also complicates the discrimination among the *Philaenus* species, including *spumarius* and *italosignus*. *Philaenus italosignus* shares all six morphs available in Italy with the corresponding six of *P. spumarius*, but not the other seventeen [40].

The polymorphism also suggests the need for a molecular tool to aid in the identification of *Philaenus* by non-entomologists. However, a trained entomologist can identify *Philaenus* males in about 15 min using a medium-quality stereoscope, but not females [8].

*Philaenus* species identifications can significantly benefit from molecular techniques, particularly in cases involving a large number of identifications at the species level, as seen during Xf monitoring programs [41].

The presence of *X. fastidiosa* in southern Italy has led to intensive monitoring of *P. italosignus* in both olive and non-olive orchards to assess the presence and impact of the transmitted plant pathogen in the area [4,42]. Accurate identification of vectors, males, and females is therefore necessary to develop effective monitoring and vector control [43] or infection management [44]. We note that recent advances in molecular techniques have led to improvements in the specificity of identifying *P. italosignus* [44].

We propose a new species-specific biomolecular identification test based on a highly selective and diagnostic locked nucleic acid (LNA) probe. This technology leverages the enhanced sensitivity and specificity of LNA probes, which have shown significant potential in the qPCR detection of specific targets (diagnosis, identification of genes or gene fragments, etc.) due to their high binding affinity and stability [45,46]. The chemical structure of LNA probes confers excellent resistance to nucleases, thereby enabling more accurate and sensitive analysis than traditional probes [47,48,49,50,51,52]. Using LNA probes with real-time PCR techniques has significantly improved the sensitivity and specificity in detecting tobacco mosaic viruses, grapevine phytoplasmas, plant viruses [53], and insects [54,55]. Moreover, as reported in Rizzo et al. [55], qPCR assays with LNA probes enable the detection of *Agrilus anxius* Gory, 1841 (Coleoptera: Buprestidae) from samples containing as few as 6,4 fg/µL of the target DNA. The LNA probe provides a practical opportunity to quickly mass-identify *X. fastidiosa* candidates or vectors, regardless of their gender or instar. The array of advantages was not previously available at this level of confidence. The LNA better assesses the vector census in field application of the Integrated Pest Management Decision Support System (IPM-DSS) [56] for routine life table and survivor analysis, in view of the Xf-induced disease surveillance. Finally, accessible tools such as the probe we propose can help improve invasive modelling.

## 2. Materials and Methods

### 2.1. Insect Material

The specimens of *P. italosignus* (*n* = 81, adults) come from the collections of the UNIFI team [Department of Agriculture, Food, Environment, Forest Resources and Natural Resource Sciences (DAGRI), University of Florence] and UNIBA [Department of Soil, Plant and Food Sciences (DiSSPA), University of Bari Aldo Moro]. Some specimens from the same collections had undergone a previous SYBR Green qPCR test, as conducted by Rizzo et al. [44], which also involved numerous adult and juvenile specimens (*n* = 514) belonging to 24 different species, as reported in Table 1. The identification of *Philaenus tesselatus* (*n* = 12), *Neophilaenus campestris* (*n* = 194), and *P. spumarius* (*n* = 178), the latter two being vectors of Xf, deserves particular attention.

### 2.2. DNA Extraction

We performed nucleic acid extraction from target adult insects and non-target adult and juvenile insects according to the protocol described in Rizzo et al. [44]. Duplicate DNA extractions for each sample were eluted in 100 µL of sterile nuclease-free water. The extracted nucleic acids were either used immediately for qPCR or stored at −20 °C for future use. A QiaExpert spectrophotometer (Qiagen, Hilden, Germany) estimated DNA quality and quantity by calculating the optical density ratios A_260nm_/_230nm_ and A_260nm_/_280nm_ for diluted and undiluted DNA extracts. The amplifiability check assessed the DNA suitability for qPCR testing using a qPCR probe reaction using the primers 18S uni-F/18S uni-R (5′-GCAAGGCTGAAACTTAAAGGAA-3′/5′-CCACCACCCATAGAATCAAGA-3′) and the probe 18S uni-P (HEX-ACGGAAGGGCACCAGGAGT-BHQ1), which amplifies a highly conserved region of the 18S ribosomal DNA of eukaryotes [57].

### 2.3. Analysis of Intra-Individual Variation in the qPCR Target Region of the Cytochrome B Gene

The cytochrome B (*cyt*B) mitochondrial gene served as a candidate gene for discriminating *P. italosignus* from other Aphrophoridae using quantitative Polymerase Chain Reaction (qPCR). To verify the existence of multiple mitochondrial haplotypes (heteroplasmy) and NUMTs (nuclear pseudogenes) within a single individual, primers CB-N3665 5′–GTCCTACCATGAGGTCAAATATC–3′ [58] and the novel primer *cyt*B-uni 5′–GGRATAAAATTATCAGGGTCYCC–3′ were used to amplify a fragment of 380 bp based on the *cyt*B nucleotide sequence of *P. italosignus*, see GenBank: FJ664097.1, NCBI link: https://www.ncbi.nlm.nih.gov/nuccore/FJ664097.1/ (accessed on 26 April 2023), and [11]. The reagent mix composition was 1 X GreenTaq PCR Buffer (Thermo Fisher Scientific, Waltham, MA, USA), 0.2 mM of each dNTP, 1.25 U of DreamTaq (Thermo Fisher Scientific, Waltham, MA, USA), 0.2 µM of each primer (Eurofins Genomics, Ebersberg, Germany), and ca. 10 ng of DNA extracted as indicated above from adults of *P. italosignus* (specimen Pi P1), *N. campestris* (Nc C8 and Nc G6), *N. lineatus* (Nl 7/1), *Neophilaenus* sp. (N 1/2), *P. spumarius* (Ps D6 and Ps G2), and *P. tesselatus* (Pt 2/1) identified to genus or species level based on morphological traits. Nc C8, Nc G6, Ps D6, Ps G2, and Pi P1 were sampled in Italy [44]; meanwhile, N 1/2, Nl 7/1, and Pt 2/1 were from Tunisia. Thermal cycling consisted of 3 min at 95 °C for initial denaturation, followed by 35 cycles of denaturation at 95 °C for 30 s, annealing at 56 °C for 30 s, and extension at 72 °C for 30 s; a final extension of 30 min at 72 °C followed. PCR products obtained were purified using FastAP and Exonuclease I (Thermo Fisher Scientific, Waltham, MA, USA) according to the manufacturer’s protocol and subjected to direct Sanger sequencing in forward and reverse or purified from 1% agarose (Invitrogen, Thermo Fisher Scientific, Waltham, MA, USA) gels using the ReliaPrep DNA Clean-Up and Concentration System (Promega Corporation, Madison, WI, USA), and ligated, propagated, and PCR amplified as described in Marchi et al. [59]. Three clones/specimens underwent sequencing in both forward and reverse directions using T7 and SP6 plasmid primers. Chromas (Technelisyum) visualized the chromatograms. Sequences were aligned using Muscle, as implemented in the MEGA version 12 software package [60]. The BLAST software (https://blast.ncbi.nlm.nih.gov/Blast.cgi, accessed 21 June 2023) analyzed three consensus sequences/specimens for their suitability as representatives of their respective genera. Then, BLAST translated the amino acid sequences to tentatively assign them to NUMTs [61] using the ExPASy Translate tool (https://web.expasy.org/translate/, accessed on 21 June 2023). To determine intra-individual variation in the *cyt*B fragment sequence, the number of haplotypes and the number of segregating sites were calculated using DnaSP v6 software [62]. When the homologous sequences from two clones differed by one or more nucleotides, the sequences included different haplotypes [61].

The oligos and LNA probe specific for *P. italosignus* were derived from the genomic region of cytochrome B (GenBank: accession number FJ664097.1, NCBI link: https://www.ncbi.nlm.nih.gov/nuccore/FJ664097.1/, accessed on 21 July 2025), encompassing a 170 bp genomic region (Table 2).

### 2.4. Design of P. italosignus Primers and LNA Probe

**Figure 1:**
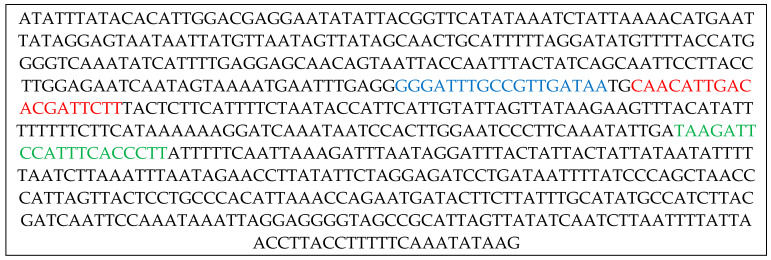
Sequence of a 650 bp fragment of the *cyt*B gene from *P. italosignus* (GeneBank: FJ664097.1), indicating the Sense Primer (in blue), the Anti-Sense Primer (in green), and the LNA probe (in red).

OligoArchitect^TM^ Primers and Probe Design Online software (Sigma-Aldrich, St. Louis, MO, USA, version 2014) facilitated the design of oligos and the LNA probe, taking into account primer melting temperatures, amplicon length, and the avoidance of secondary structures (Figure 1).

**Figure 2:**
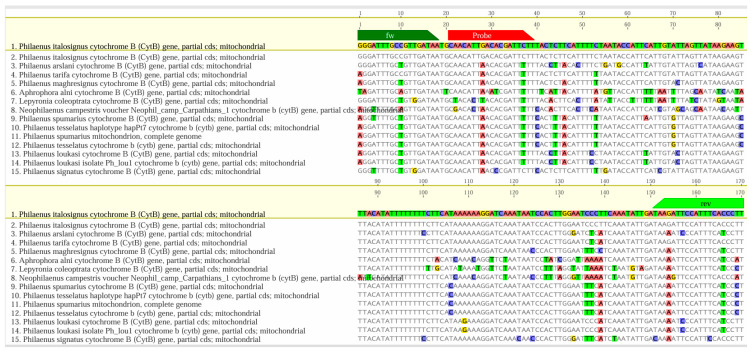
Graphical alignments between the *P. italosignus* amplicon (including primers and the newly designed LNA probe) and 15 representative sequences from some genetically related species (*Philaenus* spp., *Neophilaenus* spp., etc.). The sequences of the primers and LNA probes are in green and red, respectively. We highlighted nucleotide alignment mismatches using the following colors: blue for C (cysteine), yellow for G (guanine), red for A (adenine), and green for T (thymine). See the complete picture in Figure S1, Supplementary Material.

The software accurately determined the position of the LNA probe, covering all insects included in the study. Eurofins Genomics (Ebersberg, Germany) synthesized the oligonucleotides. At the same time, BLAST (https://blast.ncbi.nlm.nih.gov/Blast.cgi) analyzed the primers and probe design, searching for homologous nucleotide sequences and aligning them with the MAFFT software package [63], in Geneious version 2025.1.3 (Biomatters Ltd, Auckland, New Zealand; http://www.geneious.com). The alignments performed within the *cyt*B gene involved 269 sequences belonging to *Philaenus arslani* (Abdul-Nour & Lahoud, 1996)*, Philaenus maghresignus* (Drosopoulos & Remane, 2000), *P. italosignus*, *P. spumarius*, *P. tesselatus*, *P. tarifa*, *P. signatus*, *N. campestris*, *Aphrophora alni* (Fallén, 1805), and *Lepyronia coleoptrata* (Linnaeus, 1758). In Figure 2, we have simplified the alignments because many of the aligned sequences were repetitive and exhibited identical variability for the target sequence. The extended version, which includes all sequences considered, is provided in Figure S1 (Supplementary Materials).

### 2.5. qPCR Optimization

To optimize the qPCR run conditions, the optimal annealing conditions and concentrations of primers and LNA probes were determined. Primers and LNA probes were tested at concentrations of 0.2, 0.3, and 0.4 µM for primers and 0.1, 0.2, and 0.3 µM for LNA probes. A thermal gradient was established using 5 ng/µL of *P. italosignus* adult DNA, with an annealing temperature range of 52 °C to 60 °C.

Gene amplification reactions were performed in the CFX96 thermocycler (Bio-Rad, Hercules, CA, USA), considering a final volume of 20 μL. The reactions were performed in a 96-well real-time PCR plate (Starlab, Milan, Italy) with 0.2 mL wells, and each reaction was run in duplicate. We performed duplicate analyses on all samples listed in Table 1. Each cycle included a no-template control (NTC), i.e., water, as well as positive and negative amplification controls for the target samples. We repeated the test in case of unclear or contradictory results. All data were analysed using CFX Maestro software, version 2.3 (Bio-Rad, Hercules, CA, USA).

### 2.6. Performance Characteristics

The EPPO standard PM7/98 (5) [64] suggests validating the test based on analytical specificity (inclusiveness and exclusivity), analytical sensitivity, repeatability, and reproducibility. Analytical specificity was tested by comparing qPCR amplification of target and non-target samples (Table 1), using genomic DNA extracted at a final concentration of 10 ng/µL for all insects listed in Table 1. Analytical sensitivity, which determined the limit of detection (LoD), was assessed using a 1:5 serial dilution in triplicate from the genomic DNA of single adults of *P. italosignus*. The assessment range was between 5 ng/µL and 12.8 fg/µL.

QIAxpert (Qiagen, Hilden, Germany) performed all measurements using spectrophotometry technology to analyse the concentration and purity of DNA in the samples. These measurements obtained average Cq values and standard deviations (SDs) for the target species. We examined DNA extracted from eight adult *P. italosignus* in triplicate and diluted to 0.08 ng/µL. We performed the protocol at different times and by various operators to confirm reproducibility. CFX Maestro software 2.3 analysed the qPCR amplification data.

### 2.7. Blind Panel

An in-house blind test was performed for an evaluation of diagnostic specificity and accuracy on DNA extracted from 12 target and non-target insects (two *P. italosignus*, two *P. spumarius*, two *P. tesselatus*, two *N. campestris*, two *N. lineatus,* and two NTC) used at a final concentration of 5 ng/µL. The test utilized a qPCR assay with an LNA probe, and samples were received anonymously by the various operators. Two internal groups from the Phytopathology and Molecular Biology Laboratory of the Plant Protection Service in the Tuscany Region (Italy) conducted the blind panel, each consisting of two operators. DNA samples were numbered and processed in triplicate, including true positives, false positives, true negatives, and false negatives, based on the results of the blind panel. The evaluation followed the EPPO PM7/98 (5) [64] standard validation parameters.

## 3. Results

### 3.1. DNA Extraction

Nucleic acid extractions using the protocol described above [44] were all successful. The average concentrations (ng/µL) of extracted DNA and the absorbance ratios (A260/280) were within the ranges considered optimal for subsequent test validation operations (Table 3). Furthermore, the Cq values obtained in qPCR to test the amplifiability of the 18S ribosomal gene confirmed these data [57].

### 3.2. Development and Optimization of P. italosignus-Specific qPCR

**Figure 3:**
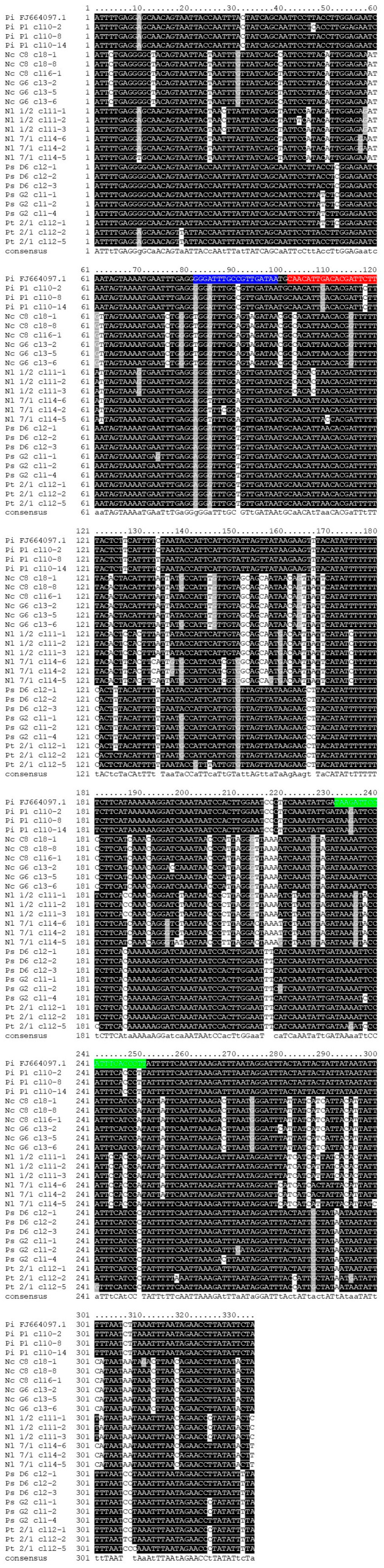
Alignment of 24 *cyt*B alleles cloned from *Neophilaenus campestris* (Nc C8 and Nc G6), *N. lineatus* (Nl 1/2 and Nl 7/1), *Philaenus spumarius* (Ps D6 and Ps G2), *P. italosignus* (Pi P1), and *P. tesselatus* (Pt 2/1) specimens used to evaluate the existence of variability due to heteroplasmy, in the nucleotide sequence targeted by the qPCR assay developed in this study. A homologous sequence (FJ664097.1) of *P. italosignus* was retrieved in the GenBank database and included for comparative purposes. The positions of the *P.italosignus*-specific qPCR assay primers (fw and rv) and probe are highlighted in blue, green, and red, respectively.

PCR amplification was performed using the *cyt*B PCR primers set CB-N3665/*cyt*B-uni to assess single-organism variability in the *cyt*B regions of interest for qPCR primers and probes. A fragment of the expected size (approx. 400 bp) was obtained from all specimens used in the trial. Although chromatograms obtained by direct sequencing had to be discarded because of superimposed mixed traces, sequencing of cloned fragments was successful and provided reliable consensus sequences. The alignment of three clone sequences for each of eight specimens (24 clones in the final dataset) showed that no gaps were present and that the number of segregating sites varied from a minimum of 1, in the cases of Ps D6 and Nl 1/2, to a maximum of 19, in the case of Pt 2/1, over 334 bp (Figure 3). Specimens Ps D6 and Nl 1/2 provided two haplotypes. Meanwhile, Nc C8, Nc G6, Nl 7/1, Ps G2, Pi P1, and Pt 2/1 provided three haplotypes. Except for Pt 2/1 clone 12-2, in whose deduced amino acid sequence an in-frame stop codon was detected (Figure 3), no other reliable evidence of NUMTs could be found, which is meaningful given that all individuals tested here possess heteroplasmy *cyt*B sequences in their mtDNA.

**Figure 4:**
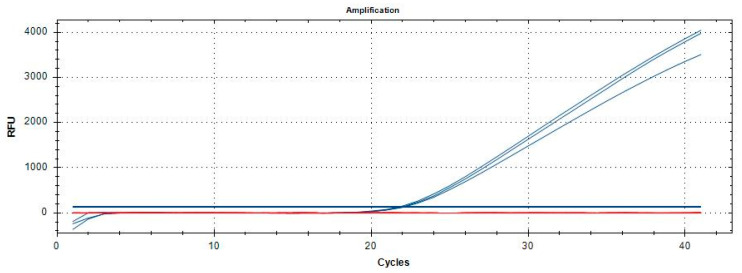
Amplification curves of *P. italosignus* adults (blue) and adults of *N. campestris*, *N. lineatus*, *P. tesselatus*, and *P. spumarius* (red). The concentrations of each extract analysed were equal to 5 ng/µL.

Optimization investigations of the qPCR assay revealed that the optimal reaction mixture (in a final volume of 20 µL) was obtained with 10 µL of 2× QuantiNova Probe PCR Master Mix (Qiagen, Hilden, Germany), using primers at 0.2 and 0.4 µM, and the LNA probe at 0.2 µM. The optimal annealing temperature for the qPCR reaction was determined to be 55 °C, as determined by thermal gradient tests. Amplification curves showing a clear inflection point, or increasing kinetics, with a Cq value < 38 strongly suggest the sample positivity. *Philaenus spumarius*, *P. tesselatus*, *N. lineatus*, and *N. campestris*, Figure 4, as well as the other samples listed in Table 1, gave no amplification.

### 3.3. Performance Characteristics

Analytical specificity tests against non-targets and targets (Table 4) yielded the expected results. The qPCR assay was inclusive for all *P. italosignus* specimens and exclusive for the non-target organisms tested. Non-target organisms had no false positives (within the reference cut-off value for the qPCR test). Therefore, both inclusivity and exclusivity resulted in an analytical specificity of 100% for the qPCR assay. Changing the analytical scenario, using different operators, extraction equipment, and volumetric dispensing equipment, etc., does not bias the qPCR assays, yielding the same qualitative results.

**Figure 5:**
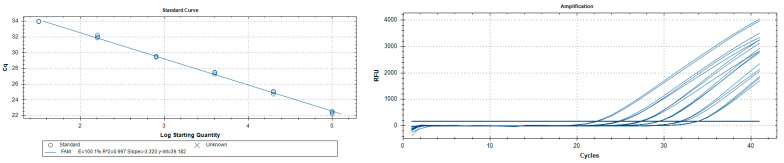
*Philaenus italosignus*: serial dilutions 1:5 of adult DNA extracts. Amplification curves are shown on the right, and titration curves on the left.

The analytical sensitivity (LoD) assessed on *P. italosignus* adults (three in number for each reaction or test thereof) was 0.064 pg/µL (Table 4) with a corresponding limit value of 37.23. The R2 correlation values for the *P. italosignus* adults analysed were 0.99, with an efficiency of 100.2% (Figure 5). Abbreviation: n/a = not applicable.

The repeatability and reproducibility of the assay are shown in Table 5 and Table 6. Eight DNA extracts (replicates) were assayed in triplicate at a concentration of 0.08 ng/µL. The range of repeatability values found was from 31.6 ± 0.15 to 32.0 ± 0.30 (Table 5), while the range for reproducibility was from 31.2 ± 0.10 to 31.7 ± 0.23 (Table 6).

### 3.4. Blind Panel

A blind test conducted in the laboratory of the Regional Phytosanitary Service of Tuscany (Italy) yielded results with 100% diagnostic specificity and accuracy, with no false positives or false negatives in the various analytical activities (Table 7). Furthermore, no non-specific reactions were found.

## 4. Discussion

A census of vectors or candidates helps in understanding the transmission dynamics of *X. fastidiosa* and the plant pathogen’s crucial role in Syndrome induction. A proper IPM approach will contain the spread of the pathogen, as well as model the vector’s dispersion risk [65,66,67,68,69,70,71,72,73]. IPM should focus more on infection management efficacy, mitigating the impact of *X. fastidiosa* [74]. Integrated Transmission Management (ITM) should prioritise the timing, triggering, and sorting of control actions [67], aiming for a more preventive and protective posture, rather than a too-late vector annihilation. ITM should combine genetic, chemical, biological, agronomical, and physical control means and actions [75,76,77,78,79,80] to minimise interactions between the quickly and LNA mass-scrutinized and identified vectors, the pathogen, and susceptible plants, thereby reducing the number of infections and the consequent acquisition opportunities for subsequent broods of the vectors. This LNA assay is significantly more sensitive than the previous SYBR Green assay, successfully detecting *P. italosignus* in larger vector or candidate mixed samples.

The morphological α-taxonomic primary identification must facilitate correct molecular identification for broader use and informed plant–pathogen findings in vector populations. It is also worth noting that a molecular test for identifying insect vectors will be used in multiple surveys to determine vector species and diagnose *X. fastidiosa* simultaneously. Finally, having a molecular identification tool can be helpful in all cases where entomological expertise for reliable and accurate recognition is lacking. It should be considered that cross-checking morphological, α-taxonomic, and molecular vector identification with *X. fastidiosa* molecular identification is a desirable strategy to follow in all cases of official activities at the territorial level by competent authorities, as it improves the reliability of the data and increases the robustness of the dataset.

Based on the scientific literature, there appears to be only one molecular protocol for the molecular identification of *P. italosignus* [44], developed using SYBRGreen technology. The need for additional molecular tools for the identification of *P. italosignus* arose when considering an alternative technique with greater analytical sensitivity. The comparison between SYBRGreen and LNA suggests replacing the former with the latter just because of LNA’s better analytical sensitivity—see the validation data for the SYBR Green assay [44]. The use of different mechanisms of action and other target genes *(cyt*B) strengthens the identification through cross-molecular confirmation. The method has a greater affinity and selectivity in comparison to standard TaqMan probes. This feature will be crucial in disease diagnosis and pest identification.

The nucleic acids extraction from adult *P. italosignus* yielded the expected results, highlighting optimal concentrations, quality, and purity values for gene amplification reactions, as was the case with the previous qPCR SYBR Green assay. Regarding analytical specificity, both in terms of inclusivity and exclusivity, a clear correspondence between the target and non-target was observed, with no evidence of non-specificity or erratic curves. The comparison involves close-level relationships very similar to *P. italosignus*, including *P. spumarius*, *P. tesselatus*, and *N. campestris*. At the genus level of *Philaenus*, we confirmed that there are no cross-reactions with the target *P. italosignus*. We found no interference with the specificity of the test when analysing 396 samples (12 of *P. tesselatus*, 12 of *N. lineatus*, 178 of *P. spumarius,* and 194 of *N. campestris*). These assessments were also carried out in silico by comparing 268 sequences belonging to different genera, including *Philaenus*. The analytical sensitivity found was 0.064 pg/µL, higher than that of the other test currently available for the specific identification of the species *P. italosignus* [44], which was 0.016 ng/µL. We note that the higher analytical sensitivity found does not appear to be essential in cases of molecular identification from adult individuals, given the amount of DNA present in each specimen (usually greater than 10 ng/μL).

It should be noted that the greater analytical sensitivity observed does not appear to be essential in cases of molecular identification from adult individuals, given the amount of DNA extracted from each specimen (usually greater than 10 fg/µL). Conversely, in cases of indirect diagnosis from genetic traces (e.g., frass, exuviae, etc.), the ability to detect very low amounts of nucleic acids is crucial. The blind panel also confirmed the validation data hypothesized regarding diagnostic specificity and accuracy (100%).

Concerning the validation data obtained, the new qPCR LNA protocol appears to be suitable for assessing the presence of *P. italosignus* in the field and for its census to estimate the size of the population and its role in the spread of *X. fastidiosa*, even in scenarios that are still unknown, assuming the homogeneous presence of *Asphodelus* host plants.

On the side of alpha-taxonomic morphological female identification, the challenge is to find several cuticular morpho-functionally specialized details in the genitalia of molecularly identified females. Female genitalia details require a different status for each species, and they have been reliably and clearly imaged using a compound microscope in bright field, serving as taxonomic characters for female-specific identification by a skilled entomologist. Reliable female identification will help assess the vector census and, consequently, facilitate the integration of the dataset for life table and survivor analysis in the next step, IPM DSS [56].

## 5. Conclusions

*Philaenus italosignus* can transmit *X. fastidiosa*, but few studies have investigated its role in the spread of the bacterium. The *P. italosignus* monophagia on *A. ramosus* suggests that it plays a marginal role in the transmission of *X. fastidiosa*. However, the recent report of new strains and sequence types of *X. fastidiosa* infecting different plants in southern Italy suggests that knowledge about *Xylella* vectors should be reviewed and updated. Morphological identification remains a primary method for identifying vectors. It is essential to propose robust results at the gene sequence level that can serve as a reference for biomolecular identification investigations (such as barcoding), especially in the design of specific molecular identification or indirect diagnosis (from genetic traces) molecular assays. The use of molecular assays capable of rapidly and accurately identifying insect vectors could help initiate sustainable phytosanitary measures against vectors and early infections promptly. The molecular assay we propose is characterized by high sensitivity and, above all, analytical specificity, thanks in part to LNA probe technology (with high binding affinity, thermal stability, and mismatch discrimination ability), which can offer more reliable and reproducible identification of male and female *P. italosignus*, even from pools, with results available in approximately two hours. Therefore, the ability to distinguish *P. italosignus* from other species with similar morphologies, particularly *P. spumarius*, is essential for evaluating and optimizing the efficacy of management strategies and plant health protection at the regional and national levels. Finally, we should consider that data on the distribution and population size of *P. italosignus* can contribute to improving predictive models of *X. fastidiosa* spread, facilitating timely containment or different measures. Alpha-taxonomic female vector or candidate identification remains challenging, but the option of new probes offers opportunities for IPM-DSS to refine the life table and analysis of survivors.

## Figures and Tables

**Table 1:** List of species in the molecular diagnostic assay. RPS: Regional Phytopathological Service Tuscany (Italy).

Order	Family	Species	Place of Collection	Number of Assayed Insects	Instar
Hemiptera	Pentatomidae	*Rhaphigaster nebulosa* (Poda, 1761)	RPS—Florence	1	Adult
	Tingidae	*Stephanitis lauri* Rietschel, 2014	University of Pisa	1	Adult
	Ricaniidae	*Ricania speculum* (Walker, 1851)	University of Pisa and RPS—Pistoia	10	Adult
	Cicadellidae	*Cicadella viridis* (Linnaeus, 1758)	RPS—Florence	1	Adult
		*Synophropsis lauri* (Horvath, 1897)	University of Florence	1	Adult
	Membracidae	*Stictocephala bisonia* Kopp & Yonke, 1977	University of Florence	1	Adult
	Aphrophoridae	*Philaenus spumarius* (Linnaeus, 1758)	University of Florence	178	Adult
		*Philaenus italosignus* Drosopoulos & Remane, 2000	University of Florence	76	Adult
			University of Bari Aldo Moro	5	Adult
		*Neophilaenus lineatus* (Linnaeus, 1758)	University of Bari Aldo Moro	12	Adult
		*Philaenus tesselatus* (Melichar, 1899)	University of Bari Aldo Moro	12	Adult
		*Neophilaenus campestris* (Fallén, 1805)	University of Florence	194	Adult
	Cercopidae	*Lepyronia coleoptrata* (Linnaeus, 1758)	University of Florence	3	Adult
		*Cercopis sanguinolenta* (Scopoli, 1763)	University of Florence	3	Adult
		*Cercopis vulnerata* Rossi, 1807	University of Florence	3	Adult
	Dictyopharidae	*Dictyophara europaea* (Linnaeus, 1767)	RPS—Florence	1	Adult
	Aleyrodidae	*Dialeurodes citri* (Ashmead, 1885)	RPS—Florence	1	Juvenile
Lepidoptera	Crambidae	*Cydalima perspectalis* (Walker, 1859)	RPS—Florence	1	Larva
	Tortricidae	*Grapholita molesta* (Busck, 1916)	University of Florence	1	Adult
		*Cydia pomonella* (Linnaeus, 1758)	University of Florence	1	Adult
		*Cryptoblabes gnidiella* (Millière, 1867)	University of Florence	1	Adult
Diptera	Tephritidae	*Ceratitis capitata* (Wiedemann 1824)	University of Florence	2	Adult
				2	Larva
		*Rhagoletis cerasi* (Linnaeus, 1758)	University of Florence	1	Pupa
		*Rhagoletis completa* Cresson, 1929	RPS—Florence	1	Larva
		*Acanthiophilus helianthi* (Rossi, 1794)	University of Pisa	1	Adult

**Table 2:** List of the primers and probes used in the assay. LNA bases of probes are in capital letters, and standard DNA bases are in lowercase.

Name	Sequence	Amplicon Size (bp)	Reference Sequence
Pitalo_63F	GGGATTTGCCGTTGATAA	170	FJ664097.1
Pitalo_232R	AAGGGTGAAATGGAATCTTA		
Pitalo_83P	FAM—caa{C}at{T}ga{C}ac{G}attctt—BHQ1		

**Table 3:** Average concentration, quality, and cycle number of crossing quantity (Cq) value of Philaenus italosignus adults. Values are means ± standard deviation.

Parameter	Adults
Concentration (ng/µL)	36.00 (±1.80)
Quality (A_260nm_/A_230nm_)	1.92 (±0.24)
Cq value	17.46 (±1.36)

**Table 4:** *Philaenus italosignus* analytical sensitivity (LoD) assays using 1:5 serial dilutions (from 5 ng/µL to 12.8 fg/µL) in triplicate (A, B, and C). The Cq values are the mean of the three threshold cycles of each dilution; Cq values above 38 were considered negative results.

Concentration	A	B	C	Average Cq	SD (±)
5 ng/μL	21.94	22.06	21.66	21.89	0.21
1 ng/μL	24.41	24.54	24.21	24.39	0.17
0.2 ng/μL	26.73	26.92	27.06	26.90	0.17
0.04 ng/μL	29.06	28.95	28.93	28.98	0.07
0.008 ng/μL	31.68	31.47	31.33	31.49	0.18
1.6 pg/μL	33.33	33.37	33.40	33.37	0.04
0.32 pg/μL	34.91	35.24	35.95	35.37	0.53
0.064 pg/μL	38.59	34.80	38.31	37.23	2.11
12.8 fg/μL	n/a	n/a	n/a	n/a	n/a

**Table 5:** *Philaenus italosignus*: the assay’s repeatability values (average and SD) in triplicate (A, B, and C).

ng/μL	Replicas	1	2	3	4	5	6	7	8
0.008	A	32.08	31.54	31.91	32.05	32.10	31.98	32.03	31.89
	B	32.13	31.72	31.55	31.77	31.78	31.66	32.24	31.77
	C	31.61	31.43	31.60	31.41	31.54	31.33	31.64	31.46
**Average Cq**		31.9	31.6	31.7	31.7	31.8	31.7	32.0	31.7
	**SD**	0.29	0.15	0.20	0.32	0.28	0.33	0.30	0.22

**Table 6:** *Philaenus italosignus*: the assay’s reproducibility values (average and SD) in triplicate (F, G, and H).

ng/μL	Replicas	1	2	3	4	5	6	7	8
0.008	F	31.61	31.28	31.37	31.71	31.79	31.41	31.86	31.29
	G	31.46	31.21	31.42	31.63	31.60	32.06	31.47	31.72
	H	31.39	31.41	31.94	31.72	31.41	31.63	31.47	32.15
**Average Cq**		31.5	31.2	31.4	31.7	31.7	31.7	31.7	31.5
	**SD**	0.11	0.10	0.32	0.05	0.19	0.33	0.23	0.43

**Table 7:** Blind panel for *P. italosignus*: results; NTC: no-template control.

N.	Sample	Lab 1	Lab 2	Expected Result
1	*Philaenus italosignus*	Positive	Positive	Positive
2	*Philaenus spumarius*	Negative	Negative	Negative
3	*Neophilaenus campestris*	Negative	Negative	Negative
4	NTC	Negative	Negative	Negative
5	*Neophilaenus lineatus*	Negative	Negative	Negative
6	*Philaenus spumarius*	Negative	Negative	Negative
7	*Philaenus tesselatus*	Negative	Negative	Negative
8	*Philaenus italosignus*	Positive	Positive	Positive
9	*Neophilaenus campestris*	Negative	Negative	Negative
10	*Neophilaenus lineatus*	Negative	Negative	Negative
11	*Philaenus tesselatus*	Negative	Negative	Negative
12	NTC	Negative	Negative	Negative

## Data Availability

The data are available upon request from the corresponding author.

## Supplementary Materials

The following supporting information can be downloaded at https://www.mdpi.com/article/10.3390/insects16101014/s1. Figure S1: graphical alignments between the *P. italosignus* amplicon (including primers and newly designed LNA probe) and 15 representative sequences from some genetically related species (*Philaenus* spp., *Neophilaenus* spp., etc.). The sequences of the primers and LNA probes are in green and red, respectively. We highlighted nucleotide alignment mismatches using the following colors: blue for C (cysteine), yellow for G (guanine), red for A (adenine), and green for T (thymine).

## Abbreviations

The following abbreviations are used in this manuscript:

LNA	Locked Nucleic Acid
IPM	Integrated Pest Management
DSS	Decision Support System
qPCR	Quantitative real-time PCR
Xf	*Xylella fastidiosa*
UNIFI	University of Florence
UNIBA	University of Bari Aldo Moro
UNIPI	University of Pisa
LoD	Limit of Detection
NTC	No-Template Control
Nc	*Neophilaenus campestris*
Nl	*Neophilaenus lineatus*
Ps	*Philaenus spumarius*
Pt	*Philaenus tesselatus*
Pi	*Philaenus italosignus*
SD	Standard Deviation
OQDS	Olive Quick Decline Syndrome
Cq	Cycle of quantification
dNTP	DeoxyNucleotide TriPhosphates
NUMTs	Nuclear Mitochondrial DNA Segments
ITM	Integrated Transmission Management
