# Supplementary material for: Rapid Detection of Philaenus italosignus Drosopoulos & Remane, 2000 (Hemiptera: Aphrophoridae) with Real-Time PCR Probe LNA Technology
Source: Insects. 2025 Sep 30;16(10):1014. doi: 10.3390/insects16101014 (PMC12565103; doi:10.3390/insects16101014)
Supplement: Supplementary file 1 [file insects-3814579-supplementary.pdf › insects-3814579-supplementary.pdf]

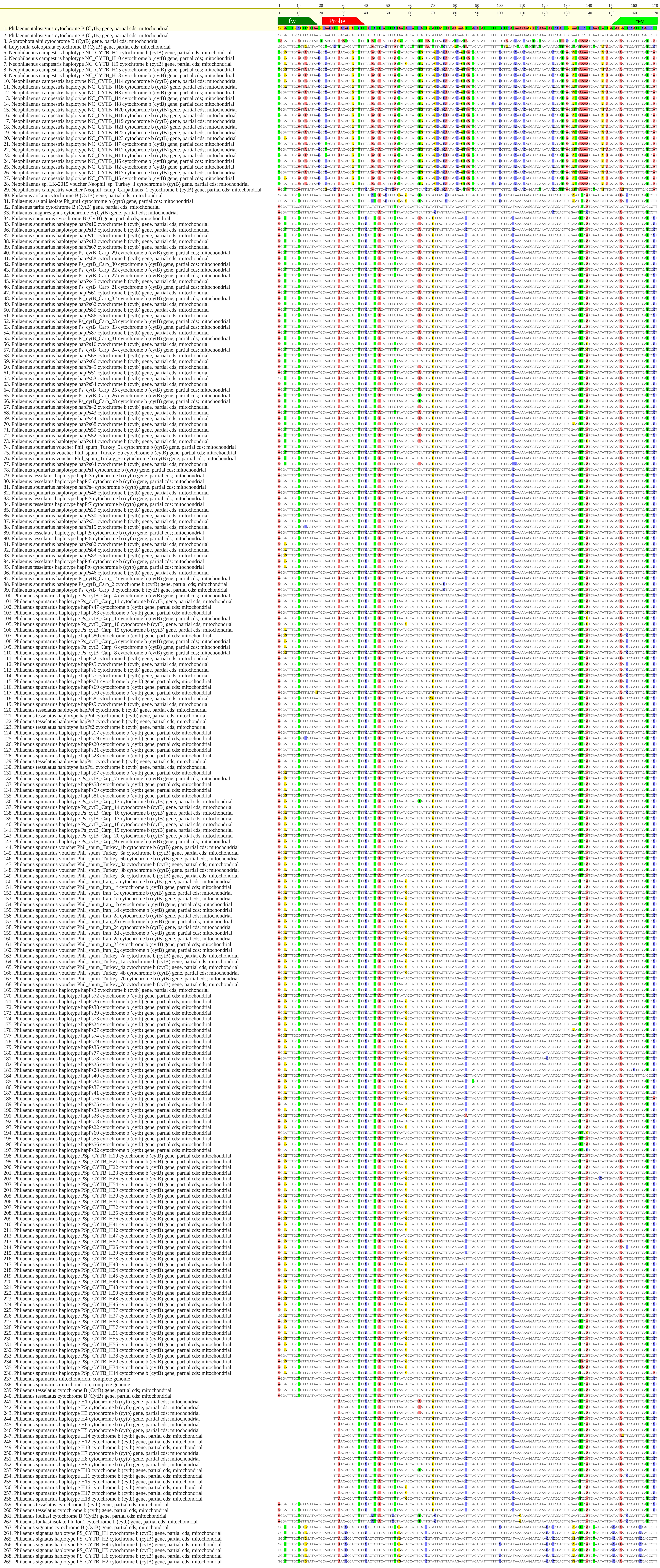
fw
Probe
rev
1. Philaenus italosignus cytochrome B (CytB) gene, partial cds; mitochondrial
